# Case Report: Catheter-based mechanical thrombectomy using the Indigo aspiration system in a case of systemic-to-pulmonary shunt thrombosis

**DOI:** 10.3389/fped.2023.1114044

**Published:** 2023-01-26

**Authors:** Natalie Soszyn, Gareth J. Morgan, John S. Kim, Jenny E. Zablah

**Affiliations:** Heart Institute, Children's Hospital Colorado, Division of Cardiology, Department of Pediatrics, University of Colorado Denver, Aurora, CO, United States

**Keywords:** case report, catheter-based, central shunt, mechanical thrombectomy, thrombosis

## Abstract

A 53-day-old girl with absent right atrioventricular (AV) connection, malposed great vessels, and pulmonary atresia underwent placement of a central shunt on the sixth day of her life. Her postoperative course was complicated by progressive desaturation, and computed tomographic angiography (CTA) demonstrated near-complete occlusion of her left pulmonary artery (LPA). Angiography demonstrated a nonocclusive thrombus in the distal central shunt and a thrombus with complete occlusion of the LPA. The Indigo aspiration system (Penumbra) was used to remove the thrombus from the central shunt and LPA, allowing placement of a stent in the narrowed LPA. Subsequent angiography showed a wide patient central shunt and LPA. The Indigo aspiration system (Penumbra) provides a viable option for removing thrombus in a patients refractory to other methods.

## Introduction

In patients with cyanotic congenital heart disease who are unable to undergo complete primary repair, staged palliation by initially placing a systemic-to-pulmonary arterial shunt is a standard method of providing a temporizing source of pulmonary blood flow (1). Unfortunately, shunt thrombosis remains a common, devastating, and often fatal complication, occurring in approximately 8%–12% of patients (1, 2). The causality of shunt thrombosis in these patients is multifactorial and remains the subject of ongoing debate (3).

The options for managing shunt thrombosis include thrombolytic therapy and systemic anticoagulation, which carry a high risk of bleeding complications, especially in young infants (4). Catheter-based interventions to recanalize the shunt and pulmonary arteries in this subgroup of patients have been widely described (5). Catheter-based endovascular mechanical thrombectomy may allow direct removal of a thrombus from a shunt without the risks associated with systemic anticoagulation and chemical thrombolysis.

We describe the case of a 53-day-old girl with central shunt thrombosis in the setting of pulmonary atresia where novel use of the Indigo aspiration system (Penumbra) allowed safe and effective removal of a thrombus from her central shunt and left pulmonary artery facilitating stent placement in her narrowed left pulmonary artery.

## Case description

**Figure 1:**
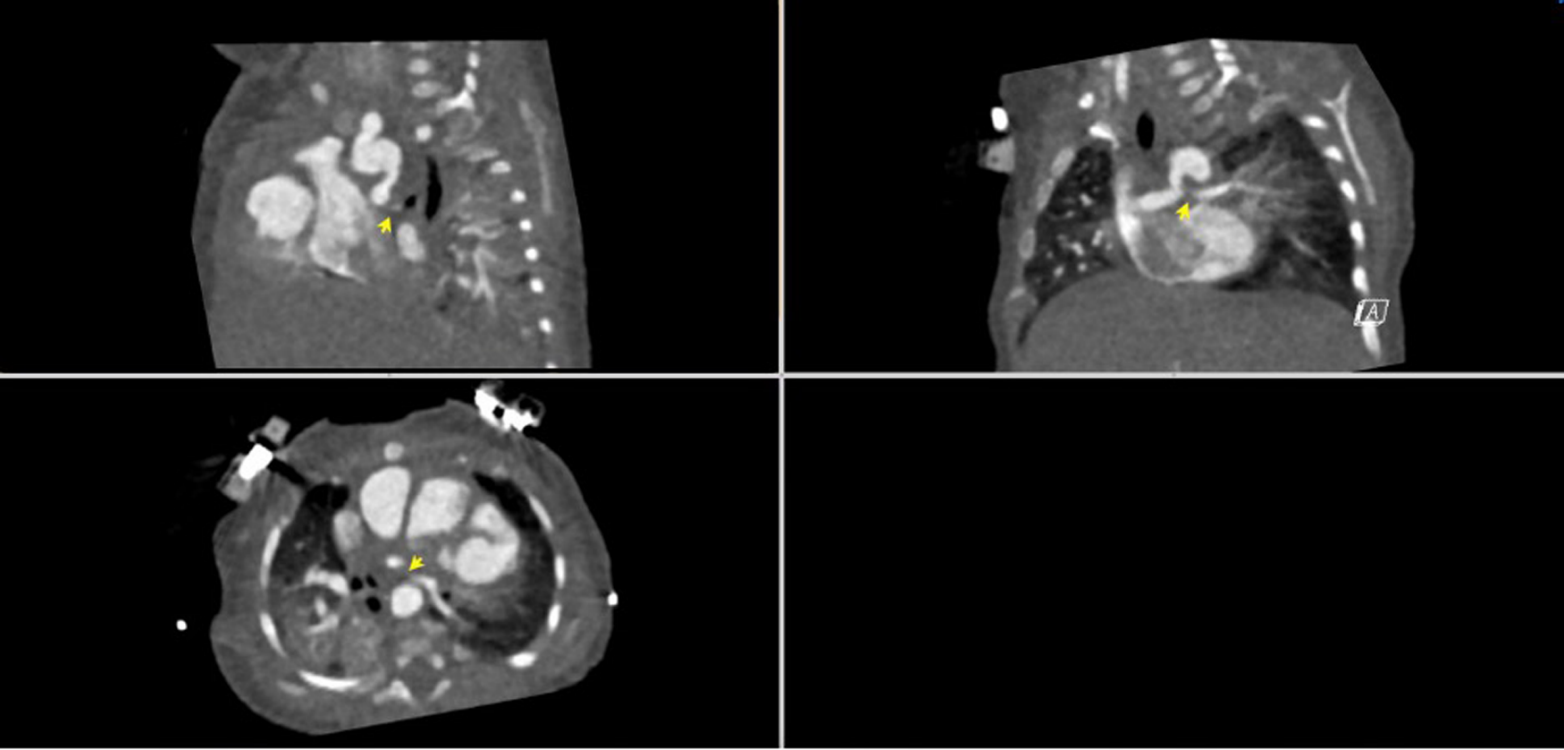
CT angiogram demonstrating the aorta-to-pulmonary central shunt that originates from the under-side of the distal transverse artic arch immediately underneath the origin of the left subclavian artery. The proximal LPA is not visualized. The LPA reconstitutes distally and is diffusely hypoplastic.

A 53-day-old, term 3-kg girl with a history of absent right AV connection, malposed great vessels, and pulmonary atresia underwent stage I palliation with a 3.5-mm aortopulmonary polytetrafluoroethylene (PTFE) graft shunt from the proximal aortic arch to the main pulmonary artery, atrial septectomy, and bilateral branch pulmonary artery patch augmentation on the sixth day of her life. Her postoperative course was complicated by ectopic atrial tachycardia, for which she was commenced on digoxin, and desaturation secondary to multilevel airway obstruction with mild supraglottic edema, laryngomalacia, and left vocal cord paralysis. As part of her assessment for low oxygen saturation, a CT scan was performed and demonstrated near-complete occlusion of her LPA and narrowing of her left main stem bronchus (Figure 1). Following multidisciplinary discussion with cardiovascular surgeons, the decision was made for the patient to undergo cardiac catheterization to recanalize the shunt and LPA and place a stent in the narrowed LPA.

**Figure 2:**
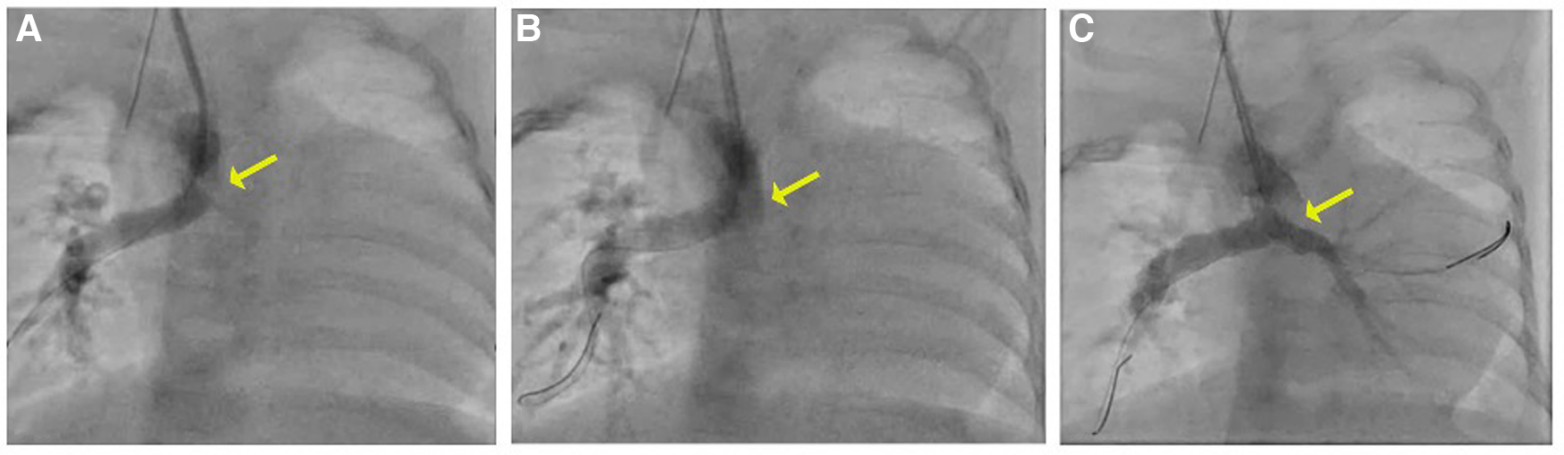
(**A**) Angiogram with a 4 Fr Glide catheter via the right carotid artery sheath demonstrates thrombus in the mid-to-distal central shunt (yellow arrow) and no contrast seen in the LPA. (**B**, **C**) On serial angiography following mechanical thrombectomy, the area of lucency is no longer present and there is improved flow in the LPA.

Percutaneous right carotid access was obtained, and a 4-Fr sheath was inserted. A 4-Fr Glide catheter (Terumo) with a 0.035 in. Glidewire (Terumo) was used to access the central shunt. The wire was removed, and an 0.014 in. PT Graphix wire (Boston Scientific) was inserted into the 4-Fr Glide catheter (Terumo) and positioned across the RPA. An angiogram was performed, which demonstrated a thrombus extending from the left pulmonary artery into the distal central shunt. Multiple wires and catheters were used to attempt to access the left pulmonary artery but were unsuccessful in passing through the thrombus. A second wire was placed into the distal right pulmonary artery. A 4-Fr, 50-cm-long Penumbra CAT3 catheter was advanced through the right carotid artery over both wires attached to the Indigo aspiration system, and a thrombectomy was performed. Although no thrombus was detected in the filter, there was a decrease in the size of the thrombus in the central shunt and LPA on serial angiography, with new evidence of flow seen to the distal LPA (Figure 2).

A 3.3-Fr JR 1.0 catheter (Pediavascular) was placed next to the wire in the right pulmonary artery and positioned at the mouth of the left pulmonary artery. An 0.014 in. PT Graphix wire (Boston Scientific) was then placed through this catheter and positioned in the distal LPA. An angiogram was performed to confirm positioning. An 0.014 in. Grand Slam wire (Asahi Intecc Medical) was then used as the working wire over which to advance a 4 mm × 8 mm Resolute Onyx drug-eluting stent (DES) (Medtronic) and was positioned across the area of LPA stenosis. The balloon was inflated to 14 atm, and the stent was deployed in the LPA. Angiography demonstrated antegrade flow to the distal LPA with significant diameter improvement of the central shunt and the LPA.

Following the procedure, she was commenced on a continuous unfractionated heparin infusion on the night following the procedure and then transitioned to therapeutic-dose subcutaneous low-molecular-weight heparin in conjunction with aspirin until her second-stage palliation procedure. Follow-up echocardiography demonstrated a patent central shunt with improved flow through the LPA stent and no evidence of a residual thrombus or distal thrombus. Follow-up CT was not performed due to the clinical improvement of the patient following the procedure. She was discharged 6 days postprocedure with no neurological sequelae and successfully underwent second-stage palliation at 4 months of age. At the time of her second-stage palliation, the LPA was hypoplastic, and the LPA stent had evidence of previous thrombosis and profound intimal irregularities. The stent was therefore removed, and the LPA was augmented using a circular Matracell patch.

## Discussion

Shunt thrombosis remains a potential life-threatening complication of palliative arterial shunt procedures, particularly in the immediate postoperative period, with a reported prevalence of 12% and a mortality rate of 4% (6). The pathogenesis is likely complex and multifactorial and includes patient-related hemodynamic and anatomic factors in combination with extrinsic and/or intrinsic prothrombotic alterations related to the surgical procedure (3). In this case, the etiology of shunt thrombosis was likely related to similar factors, although other underlying causes were investigated as per CICU/Hematology protocols without evidence of a genetic or other evident cause for the prothrombotic state. Prompt identification and management are imperative in minimizing mortality and morbidity. Management strategies in the event of shunt thrombosis include systemic anticoagulation, surgical revision and thrombectomy, and catheter-based interventions.

Systemic thrombolysis with tissue plasminogen activator has the disadvantage of increasing the risk of bleeding complications in these already vulnerable patients. Local thrombolytic therapy has been used successfully in some infants, but the experience is limited, and extreme caution is still necessary (4, 7). Additionally, thrombolytic therapy in shunt thrombosis is often contraindicated in the immediate postoperative period, particularly in patients with evidence of shock, acidosis, coagulation disturbances, overt or occult bleeding, and brain ischemia due to high risk for hemorrhagic complications (5).

Although, historically, surgery was undertaken to manage shunt occlusion, catheter-based interventions have now been used successfully for many years (8, 9). Catheter-based interventions represent a minimally invasive option to restore shunt patency that circumvents many of the risks associated with systemic thrombolysis and reoperation (2, 5). Recanalization of an occluded shunt can be achieved by mechanical clot disruption, pharmacologic dissolution, and rheolytic catheter thrombectomy in combination, usually, with balloon angioplasty and/or stent placement (8, 10, 11).

When pharmacologic thrombolysis is not feasible or ineffective, thrombus removal using mechanical thrombectomy may be indicated. Catheter-directed percutaneous mechanical thrombectomy does not carry the same risks associated with systemic thrombolytic therapy. Mechanical thrombectomy catheters as primary or adjuvant therapy for the removal of thrombus are well described in adults and older children (8, 12). Data is limited, however, on their use in infants and small children with congenital heart disease. Two case reports describe the use of the AngioJet rheolytic thrombectomy system in children with shunt thrombosis (10, 11). This system uses high-velocity saline jets to create a Bernoulli effect for the dissociation and evacuation of the thrombus. Notably, however, it can cause fluid shift, blood loss, significant intravascular hemolysis, and bradycardia, limiting its use in infants and small children (8).

The Indigo aspiration system (Penumbra) is an endovascular mechanical thrombectomy device comprised of a continuous aspiration source attached to Indigo aspiration catheters (CAT), which have been engineered to be robust, trackable, and atraumatic. The built-in microprocessor features a proprietary thrombus removal algorithm that automatically controls a valve in the tubing to provide continuous or intermittent aspiration and provides procedural feedback via audio–visual cues. Catheters are available in a selection of sizes that provide access to different-sized distal peripheral vessels of the upper and lower extremities. Catheter choice depends on the size of the lumen of the vessel and the desired location of its use. A 4-Fr catheter was used in this case to minimize access sheath size in the carotid artery in a small infant. It has been safely and effectively used in the management of pulmonary embolism and peripheral vessel thrombus in adults and older children (13). Its benefit in the management of pulmonary embolism has been well demonstrated, but its use in the removal of thrombi in patients with surgical systemic-to-pulmonary shunts has not previously been described. In our patient, this was an easy-to-use, safe, and effective tool in removing a thrombus to recanalize a systemic-to-pulmonary shunt without significant blood loss to facilitate stent placement in the narrowed left pulmonary artery.

## Conclusions

We report the novel use of the Indigo aspiration system in the removal of a thrombus from a systemic-to-pulmonary shunt and left pulmonary artery in an infant with tricuspid atresia. The Indigo aspiration system is a safe and effective alternative for catheter-based mechanical thrombectomy that can be used in combination with current catheter-based techniques in the event of acute shunt thrombosis in patients with system-to-pulmonary shunts.

## Data Availability

The original contributions presented in the study are included in the article/Supplementary Material, further inquiries can be directed to the corresponding author.
